# Breakthrough in Marine Invertebrate Cell Culture: Sponge Cells Divide Rapidly in Improved Nutrient Medium

**DOI:** 10.1038/s41598-019-53643-y

**Published:** 2019-11-21

**Authors:** Megan Conkling, Kylie Hesp, Stephanie Munroe, Kenneth Sandoval, Dirk E. Martens, Detmer Sipkema, Rene H. Wijffels, Shirley A. Pomponi

**Affiliations:** 1 Harbor Branch Oceanographic Institute, Florida Atlantic University, Fort Pierce, FL USA; 2 Bioprocess Engineering, Wageningen University & Research, Wageningen, The Netherlands; 3 Laboratory of Microbiology, Wageningen University & Research, Wageningen, The Netherlands; 4 Faculty of Biosciences and Aquaculture, Nord University, Bodø, Norway

**Keywords:** Animal biotechnology, Cell division, Cell proliferation

## Abstract

Sponges (Phylum Porifera) are among the oldest Metazoa and considered critical to understanding animal evolution and development. They are also the most prolific source of marine-derived chemicals with pharmaceutical relevance. Cell lines are important tools for research in many disciplines, and have been established for many organisms, including freshwater and terrestrial invertebrates. Despite many efforts over multiple decades, there are still no cell lines for marine invertebrates. In this study, we report a breakthrough: we demonstrate that an amino acid-optimized nutrient medium stimulates rapid cell division in 9 sponge species. The fastest dividing cells doubled in less than 1 hour. Cultures of 3 species were subcultured from 3 to 5 times, with an average of 5.99 population doublings after subculturing, and a lifespan from 21 to 35 days. Our results form the basis for developing marine invertebrate cell models to better understand early animal evolution, determine the role of secondary metabolites, and predict the impact of climate change to coral reef community ecology. Furthermore, sponge cell lines can be used to scale-up production of sponge-derived chemicals for clinical trials and develop new drugs to combat cancer and other diseases.

## Introduction

Sponges (Phylum Porifera) are key components of many benthic marine ecosystems. There are more than 9,000 described species that occur worldwide, from the intertidal to the deep sea^1^. Among the oldest metazoans, sponges have evolved a variety of strategies to adapt to different environments. Because they are sessile as adults, they have evolved sophisticated chemical systems for communication, defense from predators, antifoulants to prevent other organisms from growing over them, and to prevent infection from microbes filtered out of the water^2,3^. These chemicals interact with molecules that have been conserved throughout evolutionary history and are involved in human disease processes, for example, cell cycling^4^, immune and inflammatory responses^5^, and calcium and sodium regulation^6,7^.

Vertebrate, insect, and plant cell lines are important tools for research in many disciplines, including human health, evolutionary and developmental biology, agriculture, and toxicology. Although cell lines have been established for freshwater and terrestrial invertebrates (e.g., *Hydra*, *Caenorhabditis*), and long-term (>1 month) primary cultures have been reported for cells derived from tissues of the cnidarian *Anemonia viridis* and the shrimp *Penaeus*^8,9^, attempts to establish cell lines from marine invertebrates have been unsuccessful^9–11^.

Marine sponges, including some of the species in this study, are the source of thousands of novel chemicals with pharmaceutically relevant properties^12–14^. Supply of these chemicals is a bottleneck to development of sponge-derived drug leads: wild harvest is not ecologically sustainable, and chemical synthesis is challenging due to the complexity of many of the bioactive chemical compounds. *In vitro* production has been proposed as an option, but the lack of permanent sponge cell lines makes this option unfeasible.

Sponge cell lines could be used as models to understand the role of secondary metabolites in sponges, to use this information to develop new models for drug discovery, and to scale-up production of sponge-derived bioactive compounds for novel medicines. Cell lines of common reef sponges could also be used to quantify the effects of climate change (ocean warming and acidification) on uptake of dissolved organic material (DOM), a major component of the “sponge loop hypothesis” of carbon cycling and to test the hypothesis that coral reefs could become sponge reefs as climate changes^15^.

To date, primary cultures have been established from dissociated and cryopreserved cells of several sponge species^4,16–18^; optimized nutrient media have been developed^16,19–22^; cell division has been stimulated with growth factors and mitogens^23,24^ (Munroe *et al*. in prep); transient expression of immortalizing genes has been obtained^25^; somatic cell hybridization has been demonstrated^26^; and methods for three-dimensional culture in hydrogels have been established^27,28^ (Munroe *et al*. in prep). These improvements in sponge cell culture were sporadic and incremental, and resulted only in a limited number of cell divisions. In this study, we report a breakthrough in marine invertebrate (sponge) cell culture: using an optimized nutrient medium^22^, a substantial increase in both the rate and number of cell divisions has been accomplished for the first time.

## Results

### An amino acid-optimized nutrient medium stimulates rapid cell division in primary cell cultures of marine sponges

**Figure 1:**
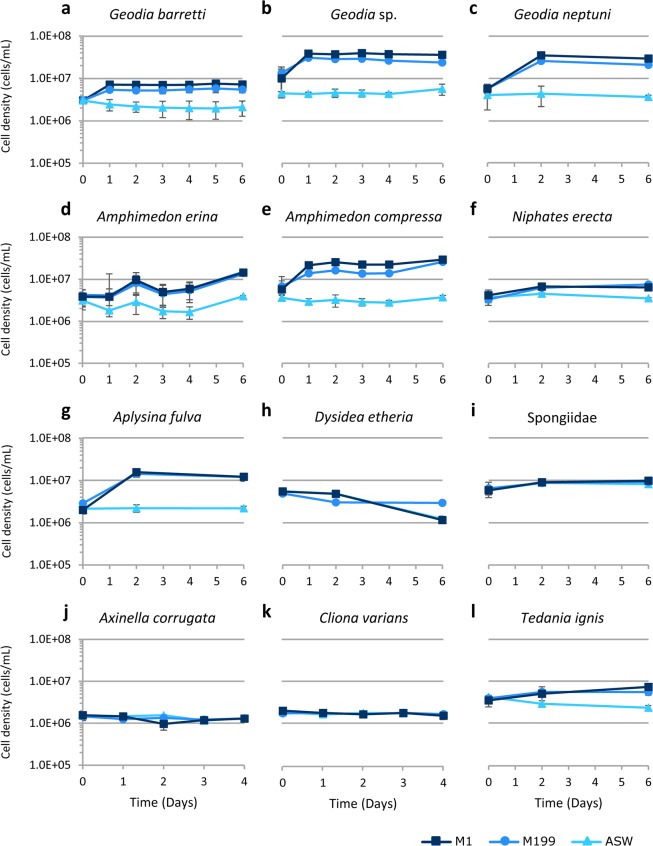
Primary cultures of 12 sponge species in three different media: artificial seawater (ASW), Medium 199 (M199) and M1 medium. (**a**) *Geodia barretti*, (**b**). *Geodia* sp., (**c**). *Geodia neptuni*, (**d**). *Amphimedon erina*, (**e**). *Amphimedon compressa*, (**f**). *Niphates erecta*, (**g**). *Aplysina fulva*, (**h**). *Dysidea etheria*, (**i**). Spongiidae, (**j**). *Axinella corrugata*, (**k**). *Cliona varians*, (**l**). *Tedania ignis*. Only 1 individual of each species was tested, however, the results are the average of 3 technical replicates (n = 3) ± standard deviation.

We cultured cells of 12 sponge species in three different media: artificial seawater (ASW), Medium 199 (M199), and M1 (Fig. 1). As predicted from prior research^4,16,17^, the number of cells cultured in ASW either remained the same or decreased, except for an unidentified species of Spongiidae (Fig. 1i). Although the pattern of cell number increase and decrease in ASW for *Amphimedon erina* parallels the pattern for M199 and M1 after the initial decrease in ASW (Fig. 1d), there was only a small increase in cell number in ASW (~25%) compared with nearly a three-fold increase in cell number in M199 and M1 after day 1. Within two days of incubation in M1, cell numbers increased for each of the following species: *Geodia barretti*, *Geodia* sp*., G. neptuni, A. erina, Amphimedon compressa, Niphates erecta, Aplysina fulva*, the unidentified species of Spongiidae, and *Tedania ignis* (Fig. 1a–g,i,l). Of these, *G. barretti*, *Geodia* sp.*, G. neptuni, A. erina, A. compressa*, and *A. fulva* had the largest increase in cell number in M1, with between 1.5 and 3 population doublings (Fig. 1a–e,g). Cell numbers also increased in M199 for the same six species, although either lower than (*G. barretti*, *Geodia* sp., *G. neptuni*, and *A. compressa*) or equal to (*A. erina* and *A. fulva*) the increase in cell numbers in M1. For three species (*D. etheria*, *A. corrugata*, and *C. varians*), there was no increase in cell number in any medium (Fig. 1h,j,k). The medium in cultures of each individual of each species with increases in cell number changed color from pale orange to dark grey, and the cells appeared microscopically to have dark inclusions (unpublished data). These changes were observed as soon as cultures increased in cell number and became increasingly darker as the cell density increased. The color change is not associated with a change in pH of the culture medium (spent medium pH: 7.8). Research is in progress to determine the cause of the color change and to characterize the cell inclusions.

### Individual (intraspecific) variation must be factored into selection of source material

**Figure 2:**
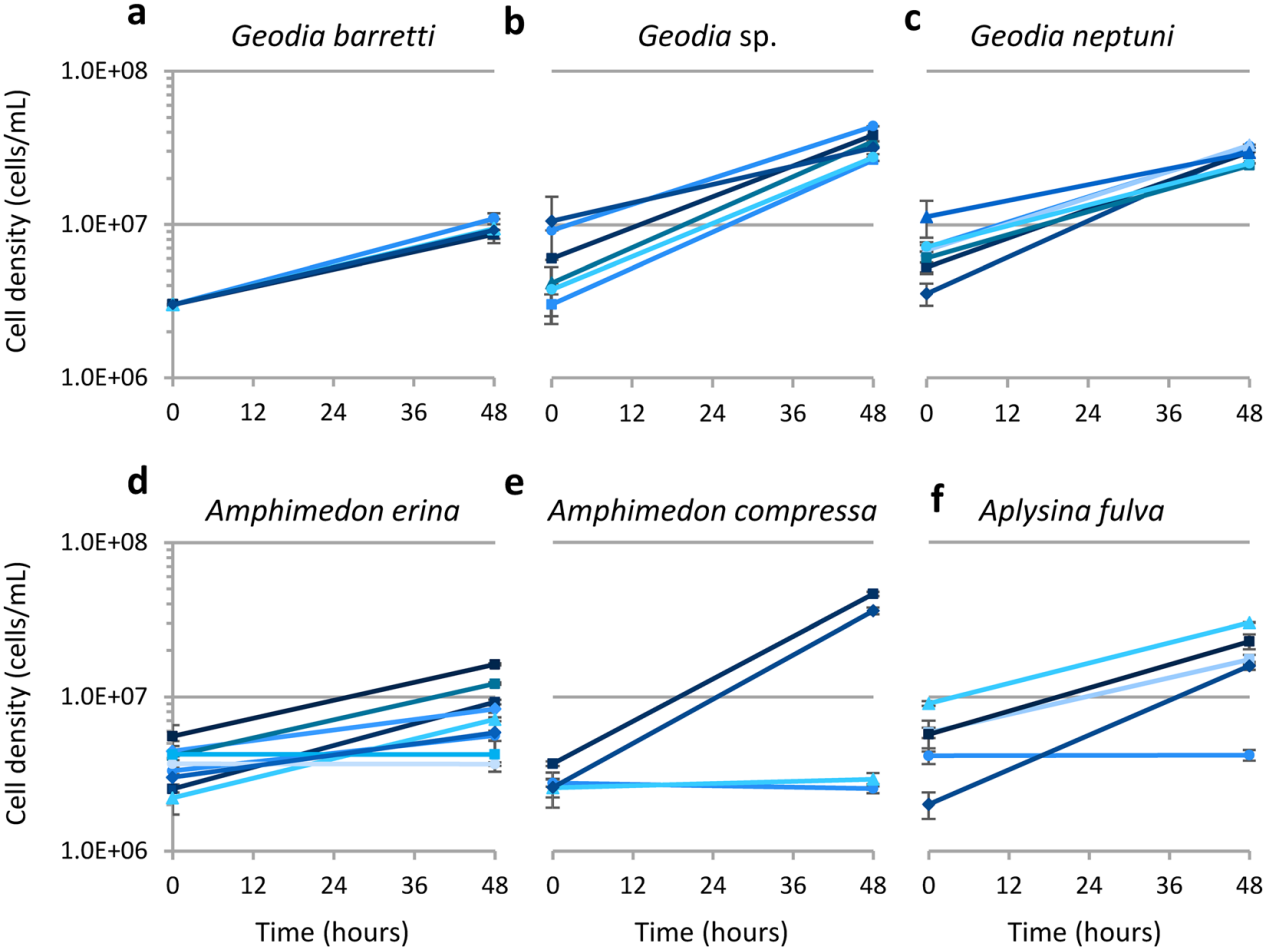
Primary cultures of six sponge species selected for further studies in M1 medium. (**a**) *Geodia barretti* (n = 4), (**b**). *Geodia* sp. (n = 6), (**c**). *Geodia neptuni* (n = 7), (**d**). *Amphimedon erina* (n = 9), (**e**). *Amphimedon compressa* (n = 4), (**f**). *Aplysina fulva* (n = 5). Each line represents an individual. Results are the average of 3 technical replicates (n = 3) ± standard deviation.

To evaluate individual (intraspecific) variation, we focused subsequent studies on the six species with the largest increase in cell density in M1: *G. barretti*, *Geodia* sp.*, G. neptuni, A. compressa, A. erina*, and *A. fulva* (Fig. 1). For these species, cells from multiple individuals were cultivated in triplicate for 48 hours in M1 medium. There was little individual (intraspecific) variation in final cell density for each of the three species of *Geodia* cultured for 48 hours (Fig. 2a–c). Conversely, individuals of *A. erina*, *A. compressa*, and *A. fulva* had individual (intraspecific) variation in final cell density (Fig. 2d–f).

### Marine sponge cells are capable of rapid cell division

**Figure 3:**
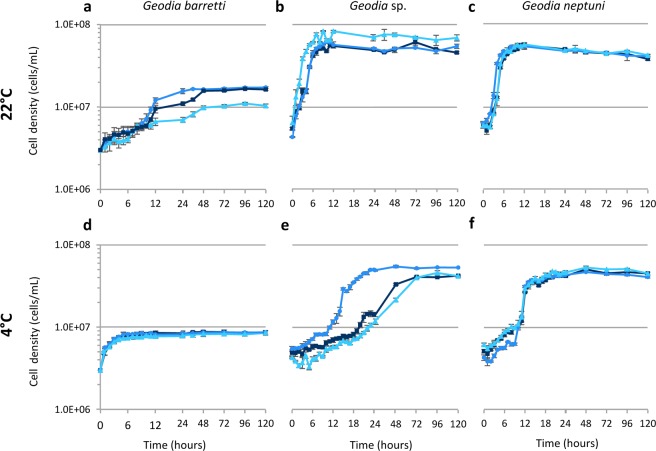
Growth curves of three species of *Geodia*. (**a–c**) 22 °C, (**d–f**) 4 °C. (**a,d**) *Geodia barretti*, (**b,e**) *Geodia* sp., (**c,f**) *Geodia neptuni*. Each line represents an individual. The results are the average of 3 technical replicates (n = 3) ± standard deviation.

In this study, we selected three species of *Geodia* because there was little intraspecific variation when cultured in M1 medium. In addition, *G. barretti* is the source of compounds with anti-inflammatory activity^29^. Cell density of *G. barretti*, *Geodia* sp., and *G. neptuni* was measured with a finer time resolution to understand the dynamics of cell division for each species (Fig. 3) and to identify when the cultures were near the end of exponential growth and, therefore, ready to subculture/passage. Growth curves were analyzed for all three species at both 22 °C (Fig. 3a–c), the temperature at which M1 medium was optimized, and 4 °C (Fig. 3d–f). These temperatures were chosen because *Geodia* sp. occurs on shallow grass flats (~2 meters) and *G. neptuni* occurs on shallow reefs (~20 meters) off Summerland Key, FL, USA, with sea surface temperatures ranging from 21.5 °C to 30.5 °C, and *G. barretti* occurs in deeper water (~500 meters) in Norwegian fjords, with sea surface temperatures ranging from 5.5 °C to 15.5 °C. Rapid cell division was observed in all *Geodia* species (Fig. 3a–f), although the number of population doublings (N_d_) varied between species and incubation temperatures (Table 1).

**Table 1:** Population doublings (N_d_) for growth curve and subculture/passaging experiments. The results are the average of 3 technical replicates (n = 3) ± standard deviation.

Experiment		*Geodia barretti*		*Geodia* sp.		*Geodia* *neptuni*	
		N_d_	σ	N_d_	σ	N_d_	σ
Growth curves	22 °C	2.26	±0.33	3.67	±0.12	3.17	±0.05
	4 °C	1.54	±0.18	3.27	±0.12	3.47	±0.17
Subcultures	22 °C	5.54	±0.17	4.67	±0.26	4.20	±0.08
	4 °C	6.95	±0.63	5.50	±0.28	5.53	±0.12

*Geodia barretti* cultures did not reach the same density as the other two species. There was little individual variation in peak cell density for *G. barretti* when cultured at 4 °C, with an average of 8.54E + 06 cells/mL (Fig. 3a). When *G. barretti* cells were cultured at 22 °C, two individuals reached peak densities of 1.67E + 07 cells/mL and 1.73E + 07 cells/mL (Fig. 3a). One individual at 22 °C had a lower peak density of 1.11E + 07 cells/mL (Fig. 3a).

Two individuals of *Geodia* sp. cultured at 22 °C reached a peak density of 6.03E + 07 cells/mL and 6.08E + 07 cells/mL within 12 hours (Fig. 3b). The third individual cultured at 22 °C reached a significantly higher peak density (8.38E + 07 cells/mL) within 9 hours (Fig. 3b). Different individual responses were observed for *Geodia* sp. cells cultured at 4 °C. Two individuals cultured at 4 °C reached a peak density of 4.29E + 07 cells/mL and 4.71e + 07 cell/mL (Fig. 3e). At 4 °C, one individual reached a higher peak density of 5.61E + 07 cells/mL within 24 hours (Fig. 3e); this was not the same individual with the highest density at 22 °C.

Cultures of all 3 individuals of *G. neptuni* incubated at 22 °C reached an average peak density of 5.54E + 07 cells/mL within 12 hours (Fig. 3c). The average peak cell density of *G. neptuni* cells incubated at 4 °C was lower (4.97E + 07 cells/mL) (Fig. 3f).

### Cultures can be subcultured and maintained for up to several weeks

**Figure 4:**
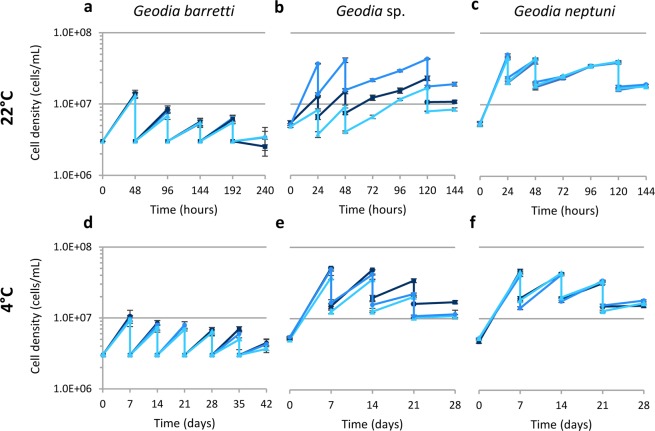
Passaging of three species of *Geodia* in M1 medium. (**a–c**) 22 °C, (**d–f**) 4 °C. (**a,d**) *Geodia barretti*, (**b,e**). *Geodia* sp., (**c,f**) *Geodia neptuni*. Each line represents an individual. The results are the average of 3 technical replicates (n = 3) ± standard deviation.

Figure 4 shows the results of subculture experiments for *G. barretti, Geodia* sp., and *G. neptuni* at both 22 °C and 4 °C. Passaging times and total number of population doublings varied among the three species. As in the growth curve experiments, *G. barretti* cells did not reach the same density as the other two species (~9.82E + 06 cells/mL at 4 °C and ~1.35E + 07 cells/mL at 22 °C) (Fig. 4a,d), however, *G. barretti* cells continued to divide until the 5^th^ passage, ultimately reaching a total of 6.95 population doublings at 4 °C and 5.54 population doublings at 22 °C (N_d_) (Table 1). Cell cultures of *Geodia* sp. had individual variations in peak cell densities when cultured at 22 °C, from 3.45E + 07 to 5.77E + 07 cells/mL (Fig. 4b). Similarly, the peak cell density of *Geodia* sp. cells cultured at 4 °C varied between individuals, from 3.79E + 07 to 5.13E + 07 cells/mL (Fig. 4e). *Geodia* sp. cells cultured at 4 °C reached a higher number of population doublings (N_d_ = 5.50) after 28 days of culture compared to cultures at 22 °C after 120 hours (N_d_ = 4.67) (Table 1). *Geodia neptuni* cultures reached 4.20 population doublings after 120 hours (5 days) (Table 1) but had an average peak cell density of 5.77E + 07 cell/mL within 12 hours (Fig. 4c). On the other hand, *G. neptuni* cultured at 4 °C had 5.53 population doublings over a course of 28 days (Table 1) and a lower average peak cell density, only reaching 4.38E + 07 cells/mL (Fig. 4f).

### Cell identity was verified by 18S rRNA gene sequence analyses

Cultures were routinely monitored microscopically, however, verification that the subcultures were the three *Geodia* species was confirmed by 18 S rRNA gene amplicon sequencing. For all samples sequenced, at least 99.96% of the reads had a 100% match with published sequence data for *Geodia* species (Table 2).

**Table 2:** 18S rRNA gene sequence match data for each species at time point zero (T0) and after four passages (P4) for *G. barretti* or one passage (P1) for *G. neptuni* and* Geodia* sp. The results are for analysis of one individual for each species.

	*Geodia barretti*		*Geodia* sp.		*Geodia neptuni*	
	T0	P4	T0	P4	T0	P4
Animalia, Porifera, Demospongiae, Tetractinellida, Geodiidae, *Geodia*	99.80%	99.84%	99.99%	99.96%	99.98%	99.94%
Animalia, Porifera, Demospongiae, Polymastiida, Polymastiidae	0.20%	0.16%	0.00%	0.00%	0.00%	0.01%
Animalia, Porifera, Demospongiae, Haplosclerida, Niphatidae	0.00%	0.00%	0.01%	0.04%	0.00%	0.00%
Animalia, Porifera, Demospongiae, Dictyoceratida, Dysideidae	0.00%	0.00%	0.00%	0.00%	0.01%	0.01%
Animalia, Cnidaria, Hydrozoa, Anthoathecata, Hydractiniidae, Clavactinia	0.00%	0.00%	0.00%	0.00%	0.01%	0.04%

## Discussion

We established finite cell lines^30,31^ for *G. barretti*, *Geodia* sp., and *G. neptuni*. The cultures were monitored microscopically and were not axenic. However, sponges are holobionts with a diverse community of microbes^32^ that may be obligate symbionts: the microbiome of *G. barretti* is species-specific and stable^33^. Continued development of sponge cell lines, and specifically, the establishment of an axenic cell line, will provide a sponge model to test hypotheses related to the functional role(s) of the sponge microbiome.

As noted, the medium in cultures with dividing cells changed color, from pale orange to dark grey, and the sponge cells appeared microscopically to have dark inclusions (~0.5 µm). The change in medium color and appearance of dark inclusions were present in each individual of each species that had increases in cell number. These changes were observed as soon as cultures increased in cell number and became increasingly darker as the cell density increased. We hypothesize that the color change is associated with the production of melanin, a photo-protective pigment that has been reported from the ectosome of marine sponges^34^. Sponge-associated bacteria also produce melanin, causing color changes to media^35^. Research is in progress in our group to determine the exact cause of the color change in the medium and to characterize the inclusions present in the cells.

Both interspecific and intraspecific (individual) variation have been observed in metabolic responses of sponges^18,22^. Over the course of this study, inter- and intra-species responses were observed for peak cell densities, number of population doublings, passaging times and culture lifespan. Cells remained in stationary phase for up to 1 week, depending on the species and the culture temperature. Our results demonstrate the necessity of testing multiple individuals of the target species to identify the appropriate individuals for continued development of cell lines. The choice of which sponge species and even which individuals to use can have a significant impact on the outcome of the study. Selecting the appropriate species and individual source material to establish marine sponge cell lines cannot be overemphasized.

The establishment of sponge cell lines requires the optimization of several variables, including nutrient media, incubation temperature, inoculation/seeding density, duration between passages, and the use of antibiotics, to name a few. Optimization of nutrient media is especially important: A genetic algorithm approach was used to optimize the amino acid composition of M199 to improve metabolic activity in primary sponge cell cultures. M1 medium was optimized from M199, based on 48-hour cultures of one sponge species, *D. etheria*^22^. Even though M1 was developed for and stimulated metabolic activity in *D. etheria*, cells from this species did not divide^22^. Nevertheless, we hypothesized that the optimized medium would stimulate cell division in other sponge species, and our results demonstrate that M1 stimulated rapid cell division in 9 other species. M199 also stimulated cell division, but M1 was better for most species. Since M1 contains extra amino acids, for some sponges the amino acid content of M199 is suboptimal. Research is in progress to optimize other medium components (e.g., lipids, vitamins, trace metals, growth factors) (Munroe *et al*. in prep) and to develop cell lines from additional species of sponges. We hypothesize that, not unlike optimization of other eukaryotic cell lines, medium optimization will be required for each species and for the intended application of the cell line.

## Conclusion

Our demonstration of exceptionally fast cell division for marine invertebrates (sponges), as well as our ability to subculture the cells, is a breakthrough in marine biotechnology. From this study, we conclude that optimization needs to be species-specific and may depend on the intended use of the cell lines. Our results form the basis for developing marine invertebrate (sponge) cell models to better understand early animal evolution and to test hypotheses related to the effects of higher temperature and lower pH on sponges. Furthermore, sponge cell lines may be used to scale-up production of sponge-derived chemicals with pharmaceutical relevance, and to gain more insight into the role of secondary metabolites in sponges to develop new models for marine natural products drug discovery.

## Methods

### Sample collection

Individuals of twelve sponge species (Class Demospongiae) from seven orders and eight families were collected for this study. *Amphimedon erina* (Order Haplosclerida, Family Niphatidae), *Cliona varians* (Order Clionaida, Family Clionaidae), *Dysidea etheria* (Order Dictyoceratida, Family Dysideidae), *Geodia* sp. (Order Tetractinellida, Family Geodiidae), an unidentified species of Spongiidae (Order Dictyoceratida), and *Tedania ignis* (Order Poecilosclerida, Family Tedaniidae) were collected from Atlantic coastal waters off Summerland Key, Florida (24°39′36.9″N 81°27′18.0″W) at a depth of approximately one to two meters, from sandy bottom grass flats and mangrove roots. *Amphimedon compressa* (Order Haplosclerida, Family Niphatidae)*, Aplysina fulva* (Order Verongiida, Family Aplysinidae)*, Axinella corrugata* (Order Axinellida, Family Axinellidae)*, Geodia neptuni* (Order Tetractinellida, Family Geodiidae) and *Niphates erecta* (Order Haplosclerida, Family Niphatidae) were collected from a deeper (~20 meters) reef site off Looe Key, Florida (24°32′44.6″N 81°24′21.4″W), characterized by hard bottom with dense sponge, coral and algae cover. *Geodia barretti* (Order Tetractinellida, Family Geodiidae) individuals were collected in a single trawl at a depth of ~500 meters in a fjord (59°58.8″N 5°22.4″E) close to Bergen, Norway. Collections from Florida were authorized under permit #FKNMS-2014-070 from the National Oceanic and Atmospheric Administration, Office of National Marine Sanctuaries, and Special Activity License SAL-14-1588-SR from the Florida Fish and Wildlife Conservation Commission.

### Sample identification

Taxonomic identification of the sponges was confirmed by evaluation of morphological characters: morphology, color, surface texture, and microscopic analysis of the skeleton. *Geodia* sp. (reported as *Geodia vosmaeri*^36^) is in the process of being re-described (Cardenas, personal communication), but it is an easily recognized species in shallow water grass flats off the coast of south Florida.

### Dissociation and cryopreservation

Cells from all studied species were dissociated and cryopreserved immediately after sampling using previously established methods^18,24,37^. Sponges were cleaned of debris and rinsed in seawater filtered through a sterile 0.22 µm filter (FSW). Cells were dissociated by squeezing fragments of sponge through sterile gauze (grade 16 mesh size for *G. barretti* [B. Braun Medical] and grade 10 mesh size for all other species [Fisherbrand]) and filtering the cell suspension through a cell strainer (40 μm [Greiner Bio-One] for *G. barretti* and 70 μm [Fisherbrand]) for all other species) to eliminate debris, cell aggregates and spicules. Cells were washed twice by centrifugation at 300 × g for 5 minutes and resuspended in FSW. Cell concentrations of *G. barretti* were microscopically counted using disposable hemocytometers (C-chip™, Neubauer improved); cells from all other species were automatically counted using the Countess II FL Automated Cell Counter (Thermo Fisher). Dissociated cells from all species were cryopreserved at a cell concentration of approximately 1.00E + 08 cells/mL in a cryoprotectant solution (10% dimethyl sulfoxide (DMSO) and 10% fetal bovine serum (FBS) in FSW)^18,24,38^. Cells were pipetted (1 mL) into cryogenic vials (Fisherbrand), the vials were placed in Nalgene^®^ Mr. Frosty freezing containers and cooled to −80 °C at a steady rate of 1 °C per minute.

### Media preparation

Artificial seawater (ASW), modified from Zhang *et al*. (2004), was prepared by dissolving salts into filter sterilized distilled water (DIW) and then autoclaving at 121 °C for 25 min^18,22^. Medium 199 (Sigma Aldrich, M3769) was prepared according to the manufacturer’s protocol. M1 medium was prepared by dissolving Medium 199 powder (Sigma Aldrich, M3769) in distilled water (DIW). Salts were added in concentrations to approximate the pH (medium: 7.9; ocean: 8.1) and salinity (medium: 33.5 ppt; ocean: 35 ppt) of seawater (Table 3). Next, amino acids were added in the concentrations that were optimized for *in vitro* culture of the sponge *D. etheria*^22^ (Table 3). Both M1 and M199 were supplemented with rifampicin (Sigma Aldrich, R3501) and amphotericin B (Sigma Aldrich, A2411) to control bacterial and fungal contamination, respectively.

**Table 3:** Composition of the three media used in this study.

Component		ASW	Medium 199	M1
		g/L		
Salts	NaCl	23.300	6.800	15.420
	Trizma HCL	4.020	—	3.769
	Trizma Base	2.970	—	2.784
	MgCl_2_	10.200	—	10.040
	Na_2_SO_4_	1.000	—	0.814
	CaCl_2_	1.000	0.200	0.400
	KCl	1.000	0.400	0.302
Amino Acids	L-Alanine	—	0.025	0.053
	L-Arginine · HCL	—	0.070	0.095
	L-Aspartic Acid	—	0.030	0.077
	L-Asparagine	—	—	0.103
	L-Cystine · 2HCl	—	0.000	0.000
	L-Cystine · HCl · H_2_O	—	0.026	0.067
	L-Glutamic Acid	—	0.067	0.114
	L-Glutamine	—	0.100	0.163
	Glycine	—	0.050	0.053
	Hydroxy-L-Proline	—	0.010	0.010
	L-Histidine · HCl · H_2_O	—	0.022	0.131
	L-Isoleucine	—	0.020	0.070
	L-Leucine	—	0.060	0.113
	L-Lysine · HCl	—	0.070	0.089
	L-Methionine	—	0.015	0.081
	L-Phenylalanine	—	0.025	0.963
	L-Proline	—	0.040	0.118
	L-Serine	—	0.025	0.869
	L-Threonine	—	0.030	0.055
	L-Tryptophan	—	0.010	0.729
	L-Tryosine · 2Na · 2 H_2_O	—	0.058	0.105
	L-Valine	—	0.025	0.128
*	Rifampicin	—	0.030	0.030
	Amphotericin B	—	0.003	0.003

*Added antibiotics and antimycotics.

### Establishment of primary cultures

For the first set of experiments, primary cultures of twelve species (one individual of each species) were evaluated to determine which species would proliferate in three different media (Table 3): artificial seawater (ASW), Medium 199 (M199)^24^ and M1 medium^22^. Cryopreserved cells were thawed rapidly in a 50 °C water bath to minimize ice crystal damage to the cells^24,37^. The cell suspension was rinsed twice by centrifugation at 4000 × rpm for 5 minutes and resuspended in ASW. Cell number was measured automatically using the Countess II FL Automated Cell Counter (Thermo Fisher) for all time points for all species except *G. barretti*, for which cell concentrations were counted microscopically using a disposable hemocytometer (C-chip, Neubauer improved). Prior to counting the cells, the cell suspension was gently pipetted to disperse aggregates. Cell aggregation was generally not an issue, however, if aggregation prevented accurate cell counts, the cells were resuspended in calcium- and magnesium-free artificial seawater (CMF)^4^ prior to counting. Cell concentrations at time point zero were calculated by counting the cell suspension and then resuspending in either ASW, M199, or M1 to the desired concentration. Samples were cultured in 24-well plates (Falcon): for the first two experiments (to determine if cells were dividing and to further evaluate cell proliferation in the 6 selected species), cells were incubated for four to six days at ~22 °C for all species except *G. barretti*, which was incubated at 4 °C. These temperatures are within the range of ambient seawater temperatures for the Florida and Norway sites, respectively. To further characterize cell division and to determine the number of times the cells could be subcultured, the three *Geodia* species were incubated at both ~22 °C and 4 °C.

### Growth characterization

Growth curves in M1 were determined for *G. barretti*, *Geodia* sp., and *G. neptuni* (n = 3 specimens for each species). Specimens (=individuals) were prepared in triplicate, in 96-well plates (Falcon), for each individual and time point. All samples were incubated at ~22 °C and at 4 °C. Cell number was measured hourly for 12 hours, every 12 hours for 48 hours and every 24 hours for 120 hours (5 days) for all cultures at 22 °C, as well as *G. barretti* cultures at 4 °C. Cultures of *Geodia* sp. and *G. neptuni* at 4 °C were measured hourly for 24 hours and every 24 hours for 120 hours. Three seeding densities (5.00E + 05, 1.00E + 06, and 5.00E + 06 cells/mL) were evaluated to determine the effect of inoculation density on final cell densities (unpublished data). The optimal seeding density (i.e., the inoculation density that resulted in the greatest increase in cell number) was 3.00E + 06 cells/mL for *G. barretti* and 5.00E + 06 cells/mL for *Geodia* sp. and *G. neptuni*. Cells were automatically counted using the Countess II FL Automated Cell Counter (Thermo Fisher) for *Geodia* sp. and *G. neptuni*, and manually counted for *G. barretti* using disposable hemocytometers (C-chip, Neubauer improved) for all time points except for *G. barretti* at time point zero, which was calculated by counting the cell suspension, and then resuspending in M1 at the desired concentration.

### Passaging cells

Cultures were passaged in M1 using three individuals per species, in triplicate, in 24-well plates (Falcon). All three species were cultured at both ~22 °C and 4 °C. The seeding density was 3.00E + 06 cells/mL for *G. barretti*, and 5.00E + 06 cells/mL for *Geodia* sp. and *G. neptuni*. Passaging was performed by resuspending the cells by pipetting, determining the cell concentration and subsequently diluting the cells, by either splitting the culture (e.g., 1:2 ratio) or diluting back to the seeding density. Cultures were passaged until the cells stopped dividing in order to determine the lifespan of the cell lines for each species. Cell number was measured automatically using the Countess II FL Automated Cell Counter (Thermo Fisher) for *Geodia* sp. and *G. neptuni*. *Geodia barretti* cells were counted microscopically using disposable hemocytometers (C-chip, Neubauer improved) for all time points except for time point zero which was calculated by counting the cell suspension and then diluting to the desired concentration. Cultures of all three species were monitored microscopically (EVOS FL Auto imaging system, Invitrogen).

### Sponge cell verification

To confirm that the cells in culture were from the three species of *Geodia*, 18 S rRNA gene amplicon sequencing and eukaryotic community profiling was performed on cultures of all three species, both before (time point zero for all three species) and after passaging (one passage, time point 48 hours, T = 22 °C for *G. neptuni* and *Geodia* sp.; four passages, time point 28 days, T = 4 °C for G*. barretti*). Cell pellets (approximately 1.00E + 08 cell/mL) of each species were stored at −20 °C. Genomic DNA extraction, 18 S rRNA gene amplification through polymerase chain reaction (PCR) and Illumina MiSeq sequencing and sequence analysis were performed by RTL Genomics (Lubbock, Texas, USA). A High Pure PCR Template Preparation Kit (Roche Life Science, Basel, Switzerland) was used to extract genomic DNA following the manufacturer’s protocol, with one exception: after addition of binding buffer and proteinase K, the samples were incubated at 70 °C for a prolonged period (35 minutes) to increase DNA yield. Fungal primers that were previously used for eukaryotic community analysis of a sponge holobiont^39^ were used to amplify an approximately 350 base pair long region of the eukaryotic small-subunit rRNA gene, including the V7 and V8 hypervariable regions in a two-step process. The forward primer was constructed with (5′-3′) Illumina i5 sequencing primer (TCGTCGGCAGCGTCAGATGTGTATAAGAGACAG) and the FF390 primer (CGATAACGAACGAGACCT)^40^. The reverse primer was constructed with (5′-3′) Illumina i7 sequencing primer (GTCTCGTGGGCTCGGAGATGTGTATAAGAGACAG) and the FR1 primer (ANCCATTCAATCGGTANT)^40^. Amplifications were performed in 25 µL reactions with Qiagen HotStarTaq master mix (Qiagen Inc., Valencia, California), 1 µL of each 5 µM primer, and 1 µL of template. Reactions were performed on ABI Veriti thermocyclers (Applied Biosystems, Carlsbad, California) under the following thermal profile: 95 °C for 5 minutes, then 30 cycles of 95 °C for 30 seconds, 50 °C for 45 seconds, and 72 °C for 1 minute, followed by a final extension of 72 °C for 10 minutes, and a 4 °C hold. Products from the first stage amplification were added to a second PCR based on qualitatively determined concentrations. Primers for the second PCR were designed based on the Illumina Nextera PCR primers:

Forward-AATGATACGGCGACCACCGAGATCTACAC[i5index]TCGTCGGCAGCGTC and Reverse-CAAGCAGAAGACGGCATACGAGAT[i7index]GTCTCGTGGGCTCGG. The second stage amplification was run with the following thermal profile: 95 °C for 5 minutes, then 10 cycles of 94 °C for 30 seconds, 54 °C for 40 seconds, and 72 °C for 1 minute, followed by a final extension of 72 °C for 10 minutes and a 4 °C hold. Sequence data were analyzed using the in-house data analysis pipeline of RTL Genomics (version 2.3.1).

## Data Availability

The datasets generated during and/or analyzed during the current study are available from the corresponding author on reasonable request.
